# The Human RAD5 Homologs, HLTF and SHPRH, Have Separate Functions in DNA Damage Tolerance Dependent on the DNA Lesion Type

**DOI:** 10.3390/biom10030463

**Published:** 2020-03-17

**Authors:** Mareike Seelinger, Caroline Krogh Søgaard, Marit Otterlei

**Affiliations:** Department of Clinical and Molecular Medicine, Faculty of Medicine and Health Sciences, Norwegian University of Science and Technology (NTNU), 7481 Trondheim, Norway; mareike.seelinger@ntnu.no (M.S.); caroline.d.sogaard@ntnu.no (C.K.S.)

**Keywords:** DNA damage tolerance (DDT), chemotherapeutics, CHK2, translesion synthesis (TLS), template switch

## Abstract

Helicase-like transcription factor (HLTF) and SNF2, histone-linker, PHD and RING finger domain-containing helicase (SHPRH), the two human homologs of yeast Rad5, are believed to have a vital role in DNA damage tolerance (DDT). Here we show that HLTF, SHPRH and HLTF/SHPRH knockout cell lines show different sensitivities towards UV-irradiation, methyl methanesulfonate (MMS), cisplatin and mitomycin C (MMC), which are drugs that induce different types of DNA lesions. In general, the HLTF/SHPRH double knockout cell line was less sensitive than the single knockouts in response to all drugs, and interestingly, especially to MMS and cisplatin. Using the SupF assay, we detected an increase in the mutation frequency in HLTF knockout cells both after UV- and MMS-induced DNA lesions, while we detected a decrease in mutation frequency over UV lesions in the HLTF/SHPRH double knockout cells. No change in the mutation frequency was detected in the HLTF/SHPRH double knockout cell line after MMS treatment, even though these cells were more resistant to MMS and grew faster than the other cell lines after treatment with DNA damaging agents. This phenotype could possibly be explained by a reduced activation of checkpoint kinase 2 (CHK2) and MCM2 (a component of the pre-replication complex) after MMS treatment in cells lacking SHPRH. Our data reveal both distinct and common roles of the human RAD5 homologs dependent on the nature of DNA lesions, and identified SHPRH as a regulator of CHK2, a central player in DNA damage response.

## 1. Introduction

Faithful replication of the genome is essential for life. DNA lesions that are not repaired prior to replication can stall replication, which may lead to mutations, replication fork collapse and genome instability. Consequences of DNA repair deficiencies are for instance carcinogenesis or neurological problems, as illustrated by the diseases Xeroderma Pigmentosum (XP), Ataxia Telangiectasia (AT) or Fanconi Anemia (FA). These diseases exhibit defects in nucleotide excision repair (NER), in the central DNA damage sensing kinase ATM or in one of the 22 FA genes important for homologous recombination and the Fanconi Anemia Pathway, respectively [1,2,3].

Cells can tolerate lesions by activating so-called DNA damage tolerance (DDT) pathways; error-prone translesion synthesis (TLS) or error-free template switch (TS) pathways after fork reversal or by strand invasion. These pathways ensure replication fork progression or restart upon replication stalling and promote the completion of DNA replication [4,5,6].

The human RAD5 homologs, helicase-like transcription factor (HLTF) and SNF2, histone-linker, PHD and RING finger domain-containing helicase (SHPRH), are multifunctional enzymes involved in TLS by activating polymerase (POL)η and POLκ, respectively [7], and in TS via polyubiquitination of PCNA [8]. HLTF and SHPRH are reported to suppress UV- or methyl methanesulfonate (MMS)-induced mutagenesis, respectively [7]. In addition, HLTF is an ATP-dependent translocase able to catalyze fork regression by its HIRAN domain [9]. Both proteins have been suggested as tumor suppressor genes, because dysregulation of HLTF and SHPRH is found in several types of cancers [10,11,12,13]. Structurally, HLTF is more similar to yeast Rad5 than SHPRH. SHPRH lacks a HIRAN domain and instead contains a histone H1 and H5 linker sequence, and a PHD domain [14]. Both RAD5 homologs bind to PCNA via their APIM sequence and this direct interaction is required to reduce the ratio of G to A (putative transcribed strand) versus C to T mutations after UV-irradiation. Furthermore, the PCNA–HLTF interaction is required for minimizing the overall mutation level [15]. Still, little is known about the exact functions and the interplay of HLTF and SHPRH in DDT.

In this study we show that the human RAD5 homologs have distinct functions in DDT, but that they are also dependent on each other. HLTF is important for minimizing the amount of mutations after both UV-irradiation and MMS treatment and SHPRH has a role in regulation of the DNA damage response (DDR) via CHK2.

## 2. Materials and Methods

### 2.1. Cell Lines

Hap1 parent cell line (Horizon) and CRISPR/Cas9-edited Hap1 HLTF^(−)^, SHPRH^(−)^ and HLTF^(−)^/SHPRH^(−)^ cell lines (HZGHC004435c010, HZGHC006910c007, HZGHC006988c001, respectively, Horizon, Cambridge, UK) were cultured in Iscove’s Modified Dulbecco’s Medium (IMDM) (ThermoFisher Scientific, Waltham, MA, USA). The HLTF ko cell line (with a 2 bp deletion in exon 2) was used to establish the HLTF/SHPRH dko cell line. The ko cell lines are all single clones. Media was supplemented with 10% fetal bovine serum (FBS) (Sigma-Aldrich, Saint Louis, USA), 2.5 µg/mL Fungizone^®^ Amphotericin B (Gibco, ThermoFisher Scientific), 1 mM L-Glutamine (Sigma-Aldrich, Saint Louis, MO, USA) and antibiotic mixture containing 100 µg/mL penicillin and 100 µg/mL streptromycin (Gibco, ThermoFisher Scientific). The cells were cultured at 37 °C in a 5% CO_2_-humidified atmosphere.

### 2.2. SupF Assay

The SupF mutagenicity assay was performed essentially as previously reported [7]. Briefly, the reporter plasmid pSP189 was irradiated with 600 mJ/cm^2^ UVB-irradiation (UV) (312 nm), with UV lamp Vilber Lourmat, Bio Spectra V5 or incubated in MMS at a final concentration of 200 mM in PBS (30 min, 30 °C). Cells were transfected with the UV or MMS (Sigma-Aldrich, Saint Louis, MO, USA) damaged reporter plasmid or an undamaged reporter plasmid as control using X-tremeGENE HP transfection reagent according to manufacturer protocol (Roche diagnostics, Basel, Switzerland). At least 3 replicas were conducted (3 Hap1 cell transfections). After 48 h the cells were harvested, and isolated plasmids were DpnI (NEB, Ipswich, MA, USA) restriction digested to exclude original bacterial plasmids in order to continue with only replicated plasmids. Isolated plasmids were transformed into *E. coli* MBM7070 cells and plated on indicator X-gal/IPTG/Amp agar plates. Blue/White screening was performed and mutation frequency (white/ blue colonies) was calculated for the different samples from multiple transformations using plasmid from the same biological replica. At least 13,000 colonies were counted from each replica. White and light blue colonies were picked for re-streaking and DNA sequencing of *SupF gene*. Colonies that did not show a mutation in the sequencing results were afterwards excluded and the mutation frequency was recalculated [15].

### 2.3. Cell Cycle and Western Blot Analysis

Cells were seeded in 10 cm dishes (220,000 cells/mL) and treated with 50 μM MMS (Sigma Aldrich) the next day. Cells were harvested for cell cycle and western blot analysis by trypsinization 12 and 24 h after treatment. Cells for cell cycle analysis cells were fixed in ice-cold methanol, washed with PBS, RNAseA-treated (100 μg/mL in PBS, 37 °C, 30 min) and DNA stained with propidium iodide (50 μg/mL in PBS). DNA staining was quantified using a FACS Canto flow cytometer (BD-Life Science, Franklin Lakes, NJ, USA) and FlowJo software. Cells for western blot analysis were lysed in 3 × packed cell volume (PCV) M-PER Mammalian Protein Extraction Reagent (Thermo Scientific, Waltham, MA, USA), PIC2 (10 μL/mL buffer) and PIC 3 (10 μL/mL buffer) (Sigma-Aldrich, Saint Louis, MO, USA) and complete protease inhibitor (20 μL/mL buffer) (Roche diagnostics, Basel, Switzerland) and incubated for 1 h at 4 °C. 1 μL Omnicleave was added to 100 μL packed cell volume (PCV). The lysate was cleared by centrifugation for 10 min at 16,000× *g*, 4 °C. Samples were run on 4–12% Bis-Tris-HCl (NuPAGE) gels. After blotting, the membrane (Immobilon PVDF, 0.2 μM) was blocked in 5% low fat dry milk in TBS (TBS with 0.1% Tween 20). The primary antibodies, CHK2-P (Thr68) (Cell signaling, 21975), CHK2 (Cell signaling, 3340), MCM2-P (S139) (Cell signaling, 8861), beta actin (Abcam, 8226), γH2AX (pSer319) (Biolegend), SHPRH (Abcam 80129), HLTF (Abcam 17984), P53-phospho (CST92845), P53 (MA5-12571) as well as the secondary antibodies IRDye 800CW (Goat Anti-Rabbit) and IRDye 700RD (Goat Anti-Mouse) Secondary Antibody (LI-COR Bioscience, Lincoln, NE, USW) were diluted in 5% dry milk in TBS and the proteins were visualized using the Odyssey Imager.

### 2.4. MTT Assay

Cells were seeded into 96 well plates (4000 cells/well) and incubated for 4 hours before treatment with methyl methanesulfonate (MMS) (Sigma-Aldrich, Saint Louis, MO, USA), mitomycin-C (MMC) (Sigma-Aldrich), cisplatin (Hospira) or UVB (Vilber Lourmat, Bio Spectra V5, 312 nm, cells exposed in 100 μL medium, additional 100 μL added after UVB exposure). The drugs were added on day 0, and cells were analyzed 24, 48 and 72 h after UVB treatment and 24, 72 and 96 h after MMC, MMS and cisplatin treatment. MTT (3-(4.5-Dimethylthiazol-2-yl)-2.5 diphenyl-tetrazolium bromide) was added to the cells and OD was measured at 565 nm, and the average from at least 6 wells was used to calculate cell survival.

## 3. Results and Discussion

### 3.1. The Two RAD5 Homologs Have Different Roles in DNA Repair or DNA Damage Tolerance Depending on the DNA Lesion

**Figure 1:**
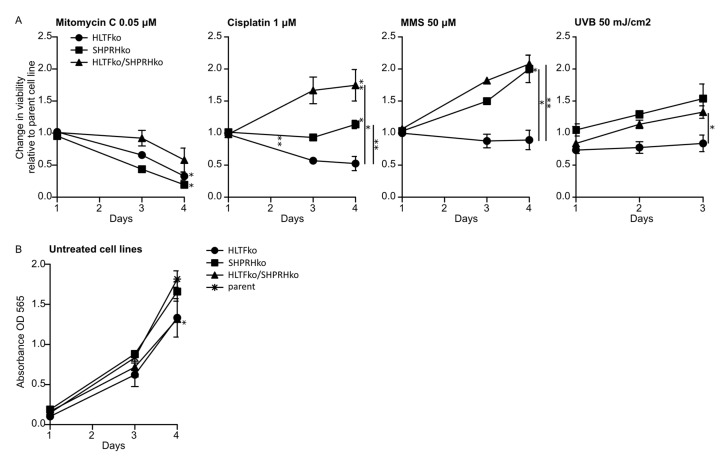
Knockouts of helicase-like transcription factor (HLTF) and SNF2, histone-linker, PHD and RING finger domain-containing helicase (SHPRH) reduces sensitivity towards several DNA damaging agents. (**A**) Viability over 3–4 days was measured in all cell lines after exposure to mitomycin-C (MMC) (0.05 μM), cisplatin (1 μM), methyl methanesulfonate (MMS) (50 μM) or UV (50 mJ/cm^2^). Change in viability in treated versus untreated ko cell normalized to similarly treated parental cells are shown, i.e., value of 1 represents the same response as in parental cells (3 independent experiments ± SD are shown). Representative parental cell response to the treatments are shown in Figure A2. (**B**) Normal growth of untreated cell lines. Two-sided student’s *t*-test, * *p* < 0.05, ** *p* < 0.01.

**Figure A1:**
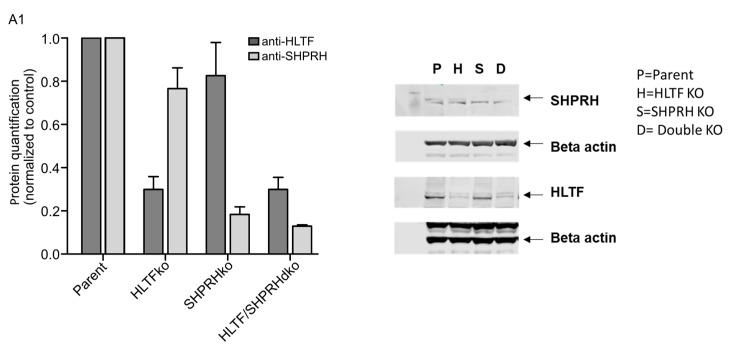
Hap1 knockout verification. Protein quantification by Western blot analysis (n = 3) (left). One representative western blot of Hap1 knockout cell lines shown (right). Arrow indicates protein of interest, SHPRH upper band, HLTF main/lower band. Related to Figure 1.

**Figure A2:**
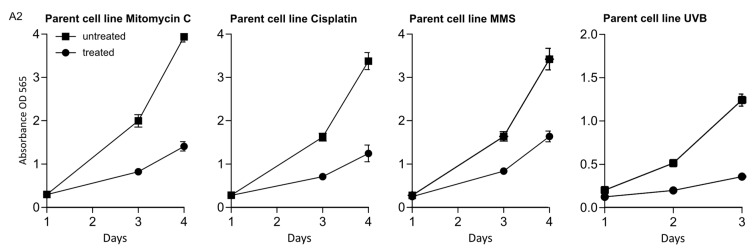
Viability of parent cell line untreated and treated with MMC (0.05 μM), Cisplatin (1 μM), MMS (50 μM) or UVB (50 mJ/cm^2^) determined by MTT on day 1, 2 and 3 (treated on day 0). Related to Figure 1.

We wanted to explore the roles of HLTF and SHPRH in DDT by using drugs which induce different types of DNA lesions. Therefore, the knockout (ko) cell lines were exposed to various DNA damaging agents, after the absence of HLTF and SHPRH expression in these cell lines was verified by western blot analysis (Appendix A, Figure A1), and treatments that gave 60–70% reduction in viability on day 3 in the parental cell line (the control) were established (Appendix A, Figure A2). In response to MMC, a reduction in cell survival was observed in all ko cell lines compared to the parent cell line. MMC is a drug which induces mainly mono-adducts and interstrand crosslinks (ICLs) with minor distortions in the DNA structure. The fraction of ICLs (~14%) produced by MMC is the main contributor to the physiological challenges after MMC treatment [16,17]. Our results suggest that both HLTF and SHPRH are important for handling MMC-induced ICLs (Figure 1A). However, interestingly, the HLTF/SHPRH double ko (dko) was less sensitive than the single ko cell lines.

In response to cisplatin the HLTF/SHPRH dko showed a significantly reduced sensitivity compared to both parent and single ko cells, while HLTF ko cells were more sensitive to cisplatin than the parent cell line (Figure 1A). Cisplatin induces mainly DNA intrastrand crosslinks (>95%), and only small amount of ICLs (2–5%) [18]. The intrastrand crosslinks formed by cisplatin are suggested to be important for cisplatin mediated cytotoxicity [16]. Thus, HLTF seems to be important for handling intrastrand crosslinks formed by cisplatin, however, not in absence of SHPRH. This indicates that HLTF and SHPRH have diverse roles and are cooperating in repair/bypass of intrastrand crosslinks and ICLs. This could indicate roles in NER and/or TLS.

In response to MMS, the sensitivity was clearly reduced in both SHPRH and HLTF/SHPRH dko cells, but HLTF ko cells were slightly more sensitive than parent cells. MMS induces mainly alkylated base lesions. The results indicate that SHPRH is not vital for handling MMS-induced alkylated lesions. This is contrary to a study that reported a SHPRH-dependent stimulation of POLκ after MMS-induced DNA lesions [7] (Figure 1A).

When examining the sensitivity to UV-irradiation, which induces cyclo-pyrimidine dimers (CPDs) and (6-4)photoproducts (6-4PPs), HLTF ko cells were again more sensitive, and SHPRH ko and HLTF/SHPRH dko cells were less sensitive than the parent cell line (Figure 1A). This supports a role for HLTF in repair/bypass of CPDs rather than 6-4PPs, because 6-4PPs are rapidly repaired by NER. This is in accordance with a report showing that HLTF stimulates TLS over UV lesions by recruiting POLη [7]. A stimulation of POLη could in addition to bypass of UV lesions, possibly also be important for cell survival after MMS, cisplatin and MMC treatment.

HLTF/SHPRH dko cells were less sensitive than the parent cells to almost all DNA lesions induced in our experiments (except DNA lesions induced by MMC). Therefore, our results suggest that cells lacking both RAD5 homologs exhibit increased DNA repair and/or DDT in response to intrastrand crosslinks, mono-adducts, CPDs, 6-4PP and alkylated bases. These lesions can be repaired by NER, BER, direct repair or bypassed by TLS. However, the dko cells did not increase DNA repair and/or DDT in response to ICLs, possibly because the repair of ICLs requires the additional activation of HR and FA pathways.

Proliferation rates in absence of treatment revealed a slightly slower growth rate of HLTF ko and dko cells (Figure 1B); however, this should only marginally contribute to the differences in the growth rates detected after treatment. In summary, cell viability measurements of the HLTF and SHPRH single ko and dko cell lines treated with different DNA damaging agents suggest that the two RAD5 homologs have both distinct functions and inter-dependent functions in mediating tolerance to different DNA lesions.

### 3.2. Absence of HLTF and SHPRH Reduces Error-Prone TLS over UV-induced DNA Lesions

**Figure 2:**
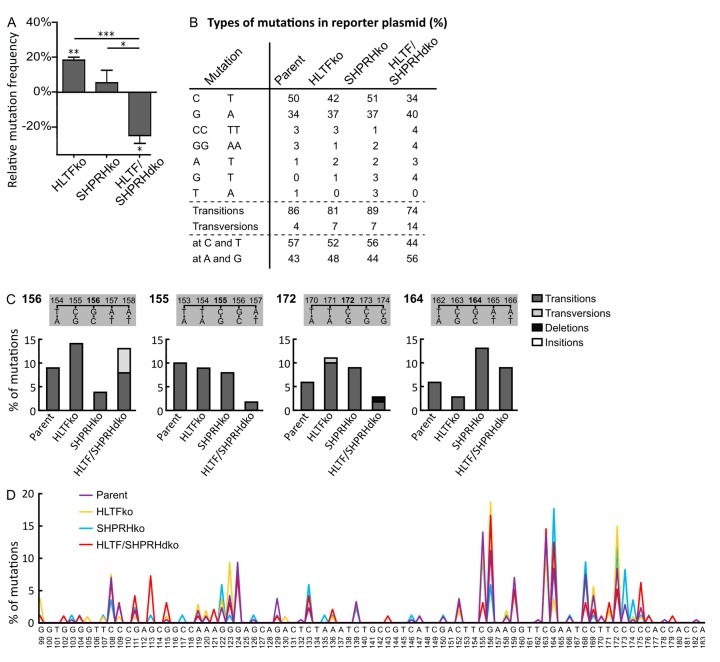
HLTF reduces error-prone DNA damage tolerance (DDT) after UVB-irradiation. (**A**) Mutation frequencies after overexpression of UVB damaged *supF* reporter plasmid pSP189 relative to control (Hap1 parent cell line) with a number of colonies counted for HLTF ko (n = 11,265), SHPRH ko (n = 25,059), HLTF/SHPRH dko (n = 13,325), Parent (n = 25,725), two-sided student’s *t*-test, * *p* < 0.05, ** *p* < 0.01, *** *p* < 0.001. (**B**) Quantification of types of mutations from sequencing mutant colonies from (**A**). Mutations with a prevalence ≥ 2% are depicted. Mutations found at T or C bases in *supF* are counted as mutations originated on the (sequenced) coding strand, mutations at A or G bases are considered to be C and T mutations originating from the transcribed strand. HLTF ko (n = 141), SHPRH ko (n = 112), HLTF/SHPRH dko (n = 127), Parent (n = 282). (**C**) Mutations at positions 155, 156, 164, 172 in *supF* gene from SupF UVB (**A**) in % with a prevalence of ≥ 10% in isolated plasmids from at least one cell line. (**D**) Mutation spectra of the *supF* gene received from sequencing mutant colonies from (**A**). HLTF ko (n = 141), SHPRH ko (n = 112), HLTF/SHPRH dko (n = 127), Parent (n = 282).

To evaluate the impact of TLS for the reduced sensitivity towards UV-irradiation and MMS exposure in cells lacking SHPRH (Figure 1A), we used a mutagenesis assay with a UV- or MMS-damaged reporter plasmid (SupF assay). However, the UV-damaged plasmids isolated from SHPRH ko cells did not show any change in the mutation frequency compared to plasmids from control cells (Figure 2A). Somewhat unexpectedly, the simultaneous absence of HLTF and SHPRH resulted in a 24% reduction in the mutation frequency, which is different to the lack of effect found previously by double knockdown of HLTF and SHPRH [7]. The reason for this discrepancy is elusive but could be due to knockdown versus knockout of the proteins. In contrast, the absence of only HLTF increased the mutation frequency in both studies (18% in our study).

Two possibilities have been suggested regarding how HLTF may reduce error-prone DDT: (i) HLTF stimulates POLη and error-free bypass of TT-CPDs [7] and (ii) HLTF induces PCNA polyubiquitination, triggering fork reversal and/or TS [8,19]. Our data from the HLTF ko cells could support both, a decreased stimulation of POLη and/or a stimulation of PCNA polyubiquitination. However, the absence of HLTF, which then possibly results in a decreased lesion bypass by POLη, seems to be “rescued” by the ko of SHPRH.

The mutation spectra of *supF* in the UV-damaged reporter plasmid isolated from HLTF/SHPRH dko cells reveal less C to T transitions (34% compared to 50% in parent cell line, 42% in HLTF ko and 51% in SHPRH ko), and more transversion mutations than plasmids isolated from the other cell lines (Figure 2B). This is further illustrated by mutations at position 155, 156 and 172 in the reporter plasmids from the different cell lines (Figure 2C). Thus, TLS across CPDs seems to be reduced in HLTF/SHPRH dko cells and instead these lesions seem to be repaired/bypassed differently. The assumption of less TLS in HLTF/SHPRH dko cells is further supported by the reduced mutation frequency in these cells. In addition, mutations at C and T bases, i.e., on the coding strand are also reduced in the absence of HLTF and SHPRH (dko cells) (44% compared to 57% in parent cells) (Figure 2B). This could theoretically be due to an increased repair on the transcribed strand and would then be an indication for a regulatory role of HLTF and SHPRH in transcription coupled repair. However, the overall reduced mutation frequency in the dko cells suggests that these cells bypass/repair UV lesions not by the “first choice” mechanisms, i.e., not by POLη or other TLS polymerases, and that these “second choice” mechanisms do not increase the mutation frequency in reporter plasmids. Polyubiquitination of PCNA can be performed by HLTF, by SHPRH, or by another unknown ubiquitin ligase [20]. Therefore, PCNA polyubiquitination might only be reduced and not completely abolished in the absence of the RAD5 homologs, and the repair of the reporter plasmids isolated from the HLTF/SHPRH dko cells could therefore still mainly be facilitated by fork reversal or TS.

In conclusion, the decreased sensitivity of dko cells towards UV-irradiation (Figure 1) is probably not mediated by increased TLS, but rather by fork reversal or TS in combination with other cellular changes such as increased repriming, increased firing of origins, increased post-replicative repair, etc. However, dko of HLTF and SHPRH could also change the balance between different TLS polymerases, and thus the balance between error-free and erroneous bypass, e.g., increase error-free bypass of 6-4PPs by POLζ [21]. The effect of HLTF ko and SHPRH ko on genome stability on chromosomal DNA can; however, not be determined by a plasmid-based assay.

### 3.3. HLTF and SHPRH Are Interdependent Proteins in Response to UV-Induced DNA Lesions

The mutation spectrum of the HLTF ko cells contains less transition mutations compared to that of the parent cells (81 versus 86%, Figure 2B), an effect that is even more pronounced in plasmids from dko cells (74%). The mutation spectrum shows different consequences of HLTF ko across identical local template sequences, e.g. at position 156 the HLTF ko resulted in an increase of mutations relative to the other cell lines, whereas at position 164 the HLTF ko resulted in fewer mutations (Figure 2C,D). The opposite applies for the SHPRH ko at the same positions. This indicates that involvement of HLTF or SHPRH in TLS across UV lesions depends on multiple mechanisms, which could be for example the distance from origins or location of the lesion (coding strand/transcribed strand, leading/lagging strand) and not only on the type of lesion and/or the sequence context.

In total, the mutation spectra analysis reveals a phenotype for the HLTF/SHPRH dko cells that differs from single HLTF and SHPRH ko cells and shows that the two RAD5 homologs are interconnected. 

### 3.4. HLTF Is Important for Correct Bypass and/or Repair of MMS-induced DNA Damage 

**Figure 3:**
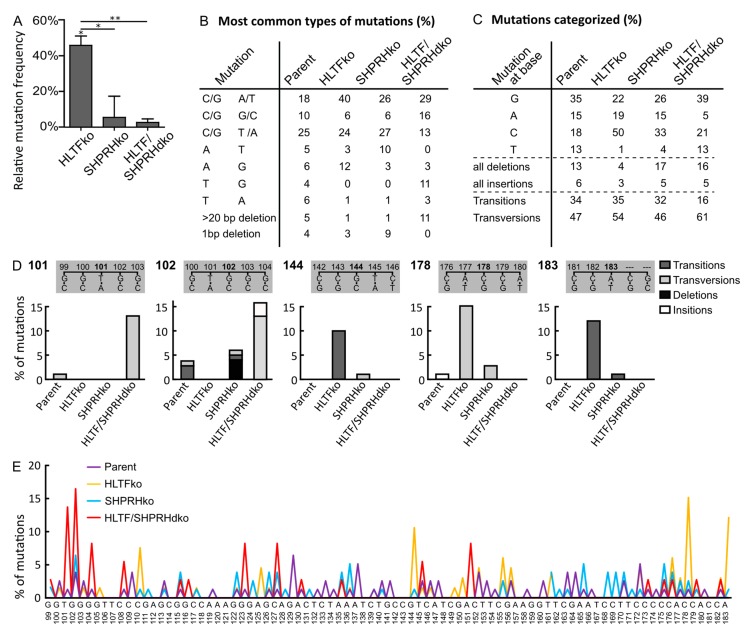
HLTF reduces error-prone DDT after MMS damage. (**A**) Mutation frequencies after overexpression of MMS damaged *supF* reporter plasmid pSP189 relative to the control (Hap1 parent cell line) with number of colonies counted for HLTF ko (n = 20,104), SHPRH ko (n = 22,294), HLTF/SHPRH dko (n = 19,130), Parent (n = 20,538), two-sided student’s *t*-test, * *p* < 0.05, ** *p* < 0.01. (**B**) Quantification of types of mutations from sequencing mutant colonies from (**A**) with a prevalence ≥5%. (**C**) Categorized mutations received from sequencing mutant colonies from (**A**). HLTF ko (n = 68), SHPRH ko (n = 78), HLTF/SHPRH dko (n = 38), Parent (n = 79). (**D**) Mutations at positions 101, 102, 144, 178 and 183 in *supF* gene from SupF UVB in % (**A**) with a prevalence of ≥ 10% in isolated plasmids from at least one cell line. (**E**) Mutation spectra received from sequencing mutant colonies from (**A**). HLTF ko (n = 68), SHPRH ko (n = 78), HLTF/SHPRH dko (n = 38), Parent (n = 79).

While plasmids isolated from SHPRH ko and HLTF/SHPRH dko cells contained an almost equal amount of mutations as plasmids isolated from parent cells, the absence of HLTF resulted in an 45% increased mutation frequency over MMS-induced lesions (Figure 3A). Thus, HLTF is important for error-free bypass of MMS-induced DNA lesions. POLη is able to bypass damages introduced by MMS, both 3meC and 3-deaza-3-methyl-2′-deoxyadenosine (a 3meA analog) in a partly correct manner [22,23]. Thus, the increased mutation frequency observed could partly be due to a lack of POLη stimulation by HLTF. In addition, the absence of HLTF likely stimulates a more error-prone TLS pathway, because HLTF is known to mediate PCNA polyubiquitination as well as strand invasion by D-loop formation [24].

Plasmids isolated from HLTF ko cells contained more C/G to A/T transversions (40% compared to 18% in parent, 26% in SHPRH ko and 29% in HLTF/SHPRH dko cells) and a general increase in mutations at C (50% compared to maximum 18% in parent cells) (Figure 3B,C). MMS causes methylations, predominantly at Gs (~83% 7meG, 1% 3meG, 16% at As, 3meA, 1meA, 7meA and less than 1% 3meC in dsDNA, reviewed in [25]). Thus, the observed increase in C/G to A/T transversions likely mainly results from lesion bypass at Gs. Error-prone bypass of 3meC could also contribute to the increased C to A transversions, because a similar pattern, i.e., increased C to A transversions and increased mutation frequency was detected in mice cells lacking ABH2, a dioxygenase which repairs 3meC (direct repair) [26]. Thus, even if 3meC is an infrequent DNA lesion after MMS in dsDNA, we cannot exclude that absence of HLTF causes a more mutagenic bypass of 3meC or that HLTF is involved in direct repair of 3meC.

Plasmids from HLTF ko cells also contained more A to G transitions (12% compared to 6% in parent, 3% in SHPRH ko and 3% in HLTF/SHPRH dko cells) (Figure 3B). Since MMS does not cause damage at Ts, these mutations likely arise from 3meA, 1meA or 7meA (16% of the lesions introduced by MMS). Known mechanisms repairing these lesions are BER and direct repair by ABH2.

### 3.5. SHPRH Has No Central Role in TLS over MMS-induced DNA Lesions, but Is Important for Avoiding DNA Strand Breaks

The mutation frequencies of MMS-treated plasmids replicated in SHPRH ko and SHPRH/HLTF dko cells were similar to the ones from the parent cell line (Figure 3A); thus, SHPRH does not seem to have a central role in TLS over MMS-induced DNA lesions.

The main change in the mutation spectrum in absence of only SHPRH (SHPRH ko) compared to the parental cell line was a doubling in A to T transversions (from 5% to 10%, Figure 3B), indicating that SHPRH could be involved in repair/error-free bypass of MMS-induced lesions in As. The mutation spectrum from SHPRH ko cells was more similar to that from the parent cells, than what was found for HLTF. Still, the mutation spectra of all cell lines revealed cell line specific patterns (Figure 3E, examples of patterns in positions 101, 102, 144, 178 and 183 are shown in Figure 3D). The most striking differences between the dko cell line and the parent cell line are a strong reduction in mutations at As (5% in HLTF/SHPRH dko compared to at least 15% in the other cell lines) (Figure 3C) and the absence of A to T transversions in plasmids from the dko cell line (Figure 3B). This suggest no/low bypass of lesions in As in the HLTF/SHPRH dko. We also detected a reduction in the total amount of transitions in HLTF/SHPRH dko cells (16%) compared to the other cell lines (32–34%) (Figure 3C).

SHPRH ko resulted in an increase in deletions; 1bp deletions in the SHPRH ko cells and larger, > 20 bp, deletions in the HLTF/SHPRH dko cells (Figure 3B). This is contrary to the reported SHPRH mediated stimulation of TLS by POLκ [7], which is reported to often cause single-base deletions [27]. The increased amount of deletions in cells lacking SHPRH, could indicate an increase in repair of strand breaks by Non-Homologous End Joining (NHEJ). Interestingly, we repeatedly isolated less replicated reported plasmid from the HLTF/SHPRH dko cells than from the other cells, and this may indicate more frequently collapsed replication forks in absence of HLTF and SHPRH. Therefore, even though the mutation frequency was not increased, our results suggest that SHPRH ko and HLTF/SHPRH dko cells have a reduced ability to handle MMS-induced lesions. The unchanged mutation frequency and increased viability after MMS treatment detected for the SHPRH ko and dko cells, does not suggest that SHPRH has a central role in reduction of TLS over MMS-induced lesions as suggested previously [7]. The reason for this discrepancy is elusive, but differently regulated DDT/DNA repair and/or DNA damage response in different cell lines and/or a lower MMS dose used by Lin et al., could potentially impact the results. In addition, knockdown versus knockout could potentially influence the levels of HLTF, or other proteins important for DDT, and thus TLS.

### 3.6. MMS Results in A More Diverse Mutation Pattern than UV

**Figure 4:**
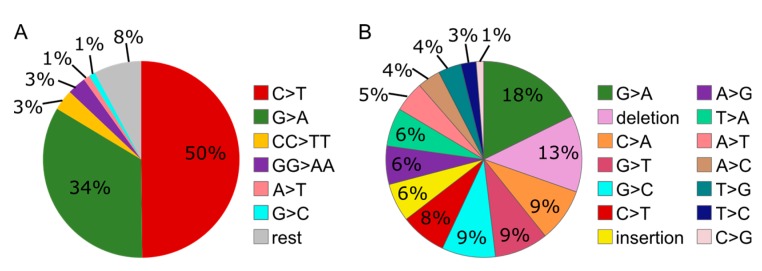
Distribution of mutation types caused by UV (control/parent only) (**A**) or MMS (control/parent only) (**B**) in the *supF* reporter plasmid replicated in control cells (Hap1 parent cell line). “Rest” accounts for mutations with a prevalence <1%.

The mutation spectra of the reporter plasmid isolated from the wild type/parent cells contained a more diverse pattern of mutations after MMS than after UV treatment (Figure 4). In addition, especially the amount of deletions was remarkably higher after MMS than UV treatment (13% compared to 0%). These results suggest a more diverse contribution of repair/bypass pathways upon MMS than UV treatment.

### 3.7. Knockout of the RAD5 Homologs Affects Cell Cycle Distribution after MMS Treatment

**Figure 5:**
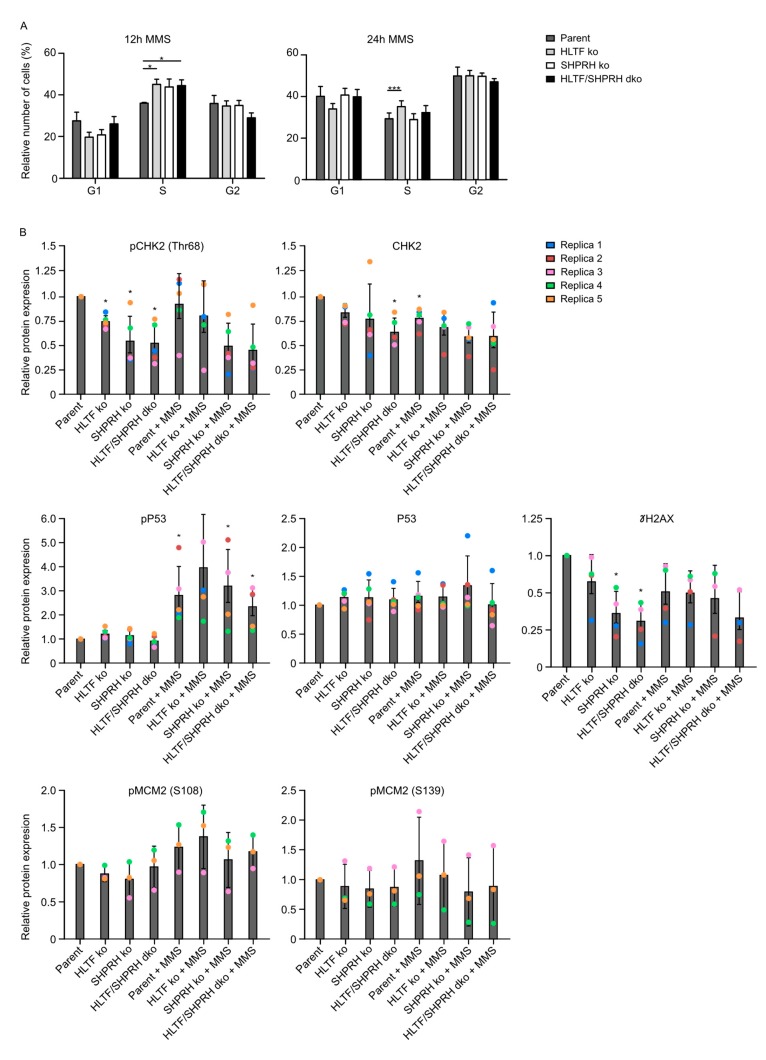
Different cell cycle distribution after MMS in ko cell lines. (**A**) Cell cycle analysis of knockout cell lines and parent Hap1 cell line 12 and 24 h after MMS treatment; 4 and 5 replica, respectively. Student’s *t*-test * *p* < 0.05, *** *p* < 0.001. (**B**) Levels of pCHK2 T68, CHK2, p53-P, p53, pMCM2-S139, pMCM2 S108 and γH2AX before and after MMS (50 μM) treatment (12 h) in different cell lines relative to the level in untreated parent cells. Level of protein detected by Western blot analysis are determined using fluorescence and loading is normalized to β-actin. One representative blot is shown in Figure A3 (Appendix A). Average ± SD as well as individual values of 3–5 replica (R1-R5) are shown. Student’s *t*-test compared to untreated parent cell line * *p* < 0.05.

HLTF ko cells were slightly more sensitive and showed an increased mutation rate, indicating increased error-prone TLS after MMS treatment, whereas SHPRH ko and HLTF/SHPRH dko cells were less sensitive and the mutation frequencies in the reporter plasmids were not increased (Figure 1 and Figure 3). The results from the viability assays and the SupF assays therefore suggest that the reduced sensitivity in absence of SHPRH is caused by other cellular DDT mechanisms than TLS. This also could include changes in cell cycle checkpoints and/or regulation of replication origin firing. Therefore, we next examined how the absence of the RAD5 homologs affects cell cycle distribution. After 12 h, all ko cell lines treated with MMS were more accumulated in S-phase compared to the parent cell line (Figure 5A), indicating a higher level of replicative stress. The important tumor suppressor p53 is phosphorylated upon DNA damage resulting in G1/S cell cycle arrest and/or apoptosis. In accordance with the accumulation in S-phase, p53 phosphorylation increased after MMS treatment; however, no difference was seen between the cell lines (Figure 5B).

After 24 h, only HLTF ko cells were marginally accumulated in S-phase, while cells lacking SHPRH did not differ much from the parent cell line, even though these cells (SHPRH ko and dko) were less sensitive to MMS.

### 3.8. Reduced Phosphorylation of CHK2 and MCM2 in Cells Lacking SHPRH

Next, we examined the activation of CHK2, a kinase important for regulating the entry into mitosis as well as phosphorylation of multiple downstream factors such as p53 and MCM2 [28,29], in ko cells treated with MMS. Interestingly, reduced levels of phosphorylated CHK2 in all ko cells were detected, with the lowest level in cells lacking SHPRH (Figure 5B). At the same time, the levels of unphosphorylated CHK2 were less reduced in the ko cell lines compared to the parent cell line. This is likely not an off target CRISPR/Cas9 effect, because the HLTF ko cells were used as the basis for establishing the HLTF/SHPRH dko and both cell lines lacking SHPRH revealed reduced CHK2 phosphorylation. Therefore, this suggests that SHPRH may directly or indirectly involved in CHK2 activation.

Twelve hours after MMS treatment all ko cells were more arrested in S-phase than the parent cell line; however, p53 phosphorylation increased similarly in all cell lines despite low CHK2 phosphorylation in the SHPRH single and dko cells. This suggests that p53 phosphorylation in these cells is not only mediated by CHK2. Further, a tendency towards a reduced phosphorylation of both MCM2 (S139) and MCM2 (S108) were detected in cell lines lacking SHPRH upon MMS treatment, i.e., the cells with lowest CHK2 activation (Figure 5B). Phosphorylation of MCM2, a component of the pre-replication complex, is needed to recruit proteins to the CMG (Cdc45-Mcm-GINS) helicase complex and activate replication.

Reduced CHK2 phosphorylation upon DNA damage is expected to result in reduced activation of the G1/S and G2/M checkpoints, followed by increased levels of collapsed replication forks and/or entrance into mitosis in the presence of unreplicated DNA [30]. However, the basal levels of γH2AX, which is a common marker for DSBs and arrested replication forks, was reduced in cells with reduced CHK2 activation (Figure 5B). This is in line with the observation that the cell lines lacking SHPRH (SHPRH ko and dko) used in our experiments were more resistant to MMS and continued to proliferate rapidly up to 4 days after treatment. Normally, in response to replication stress (late) origin firing is inhibited in presence of CHK1 and intra-S checkpoint activation [31]. We were not able to detect CHK1 phosphorylation in our western analysis (data not shown). However, CHK2, as well as other factors, might also be involved in regulation of origin firing. For example, in a recent study in yeast, inhibition of origin firing was observed in response to MMS before CHK1 was phosphorylated [32]. Interestingly, a similar phenotype, i.e., maintained replication upon MMS treatment, is reported in yeast cells with reduced Rad53-levels, the functional CHK2 homolog in yeast [33]. Furthermore, Rad53 was found to block origin firing through phosphorylation of Dbf4 [34], and in addition, Rad53 mutants contained an accumulation of reversed forks, abnormal replication intermediates and larger ssDNA regions at the replication fork than wild type yeast cells [35]. These reports support a role of CHK2 in the regulation of origin firing. If so, reduced CHK2 levels in SHPRH ko and dko cells may be responsible for the continuous proliferation observed in SHPRH ko cells after MMS treatment via activation of dormant origins, without an inhibition of late origin firing. How SHPRH knockdown reduces CHK2 activation is elusive. The reduced CHK2 activation could possibly be caused by lack of the ubiquitin ligase activity of SHPRH and increased stability of other ubiquitin ligases responsible for CHK2 turnover e.g., via SIAH2 [36], or by the recently suggested role of SHPRH as a nucleosome-E3 ubiquitin ligase, which could possibly change the gene expression level of CHK2 [37]. The ubiquitin ligase activity of SHPRH could also regulate degradation of proteins involved in activation of origin firing leading to increased origin firing independent of CHK2. Recently, ubiquitin signaling pathways were shown to be involved in the regulation of origin firing [38].

As discussed above, reduced CHK2 phosphorylation may result in a premature entry into mitosis with increased levels of stalled replications forks and un-replicated DNA/post replicative gaps. This will eventually lead to reduced genomic stability. This cannot be detected by the SupF assay used in this study; however, reduced recovery of DNA from the SupF assay in dko cells might be an indication of high levels of stalled/collapsed replication forks. However, validation of the hypothesis that SHPRH is important for regulation of CHK2 activation and regulation of origin firing requires further investigation and verifications on multiple cell lines. SHPRH might be involved in modulation/selection of multiple repair pathways. For example, MMS is an agent causing several DNA lesions that are mainly repaired by BER (reviewed in [39]); thus, the unperturbed cell growth of SHPRH ko after MMS exposure could indicate an involvement of SHPRH in modulation of BER. In yeast, Rad5 foci formation is strongly reduced after MMS treatment in *apn1* or *apn2* null mutants indicating that Rad5 recognizes BER intermediate products [40]. Excessive levels of BER intermediates, i.e., strand breaks, can potentially be toxic and/or mutagenic, thus their regulation is required in order to prevent genome instability.

## 4. Conclusions

In conclusion, in this study we show that HLTF and SHPRH have distinct properties/functions in presence of replication stress induced by different types of DNA lesions. HLTF is important for inhibiting error-prone DDT directly by stimulating error-free TLS and TS, while SHPRH is modulating DDT also via regulation of checkpoint activation.

## Figures and Tables

**Figure A3:**
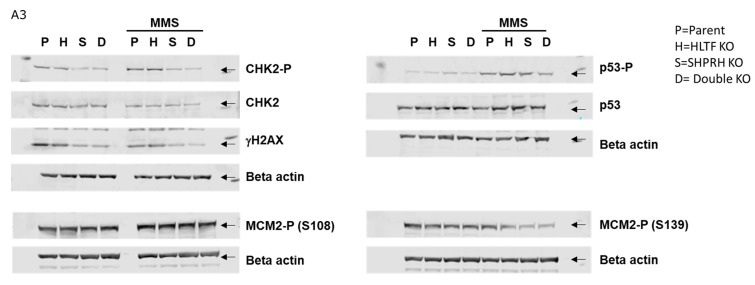
Western blot of Hap1 knockout cell lines before and after MMS (50μM) treatment (12 h), one representative out of the 3-5 biological experiments presented. Related to Figure 5. Arrow indicates protein of interest.
